# Long-term outcomes of childhood encephalitis in Australia: burden and predictors from the Australian childhood encephalitis (ACE) prospective multicentre cohort

**DOI:** 10.1016/j.lanwpc.2026.101951

**Published:** 2026-08-08

**Authors:** Gautam J. Rao, Russell C. Dale, Rebecca Burrell, Luis Furuya-Kanamori, Katherine Phan, Christopher C. Blyth, Jim P. Buttery, Julia E. Clark, Nigel W. Crawford, Helen S. Marshall, Robert Booy, Cheryl A. Jones, Philip N. Britton, Paediatric Active Enhanced Disease Surveillance (PAEDS) Network, Emma Carey, Alissa McMinn, Kate Dohle, Anna Wylie, Karen Bellamy, Guillian Hunter, Madison Bellamy, Christine Heath, Natascha D’Angelo, Carolyn Finucane, Laura Francis, Sonia Dougherty, Natasha Doran, Sara Cook, Nicole Kerly, Elizabeth Foat, Sophie Kasz, Jocelynne McRae, Nicole Dinsmore, Kathryn Meredith, Laura Rost, Shirley Wong, Joshua Francis, Jeremy Carr, Peter Richmond, Kristine Macartney, Nicholas Wood, Anne Kynaston

**Affiliations:** a Sydney Medical School, Faculty of Medicine and Health, University of Sydney, Sydney, NSW, Australia; b Department of Neurology, Sydney Children’s Hospital Network, Sydney, NSW, Australia; c Kids Neuroscience Centre, The Children’s Hospital at Westmead, Westmead, NSW, Australia; d Centre for Paediatric and Perinatal Infection Research, The Children’s Hospital at Westmead, Westmead, NSW, Australia; e The University of Sydney Infectious Diseases Institute (Sydney ID), University of Sydney, Sydney, NSW, Australia; f UQ Centre for Clinical Research, The University of Queensland, Herston, QLD, Australia; g Wesfarmers Centre of Vaccines and Infectious Diseases, Telethon Kids Institute, The University of Western Australia, Nedlands, WA, Australia; h School of Medicine, The University of Western Australia, Nedlands, WA, Australia; i Department of Infectious Diseases, Perth Children’s Hospital, Nedlands, WA, Australia; j Department of Microbiology, PathWest Laboratory Medicine, QEII Medical Centre, Nedlands, WA, Australia; k Child Health Informatics (Paediatrics), University of Melbourne, Melbourne, VIC, Australia; l Royal Children’s Hospital, Melbourne, VIC, Australia; m Murdoch Children’s Research Institute, Parkville, VIC, Australia; n Infection Management Service, Queensland Children’s Hospital, South Brisbane, QLD, Australia; o SAEFVIC, Infection & Immunity, Murdoch Children’s Research Institute, and Immunisation Service, Royal Children’s Hospital, Parkville, VIC, Australia; p Department of Paediatrics, The University of Melbourne, Parkville, VIC, Australia; q The Women’s and Children’s Health Network and the Robinson Research Institute and Adelaide Medical School, The University of Adelaide, Adelaide, SA, Australia; r National Centre for Immunisation Research and Surveillance, The Children’s Hospital at Westmead, Westmead, NSW, Australia; s The Children’s Hospital at Westmead Clinical School, Faculty of Medicine and Health, The University of Sydney, Westmead, NSW, Australia; t Department of Infectious Diseases & Microbiology, The Children’s Hospital at Westmead, Westmead, NSW, Australia; u Faculty of Medicine and Health, University of New South Wales, Sydney, NSW, Australia

**Keywords:** Encephalitis, Paediatric, Children, Long-term, Predictors, Neurological, Sequelae, Outcome

## Abstract

**Background:**

Encephalitis is inflammation of the brain parenchyma with heterogenous aetiology and presentation. In children, it often leads to short-term residual neurological deficits, however clinical predictors of adverse long-term outcome are not fully understood.

**Methods:**

Cases of suspected encephalitis, in children aged 0 to <15 years, were actively identified from five paediatric hospitals in Australia, from March 2014 to June 2018. An expert panel categorised them into aetiological subgroups. Follow-up was conducted at discharge and 12 months using the Liverpool outcome score as the primary outcome. Potential clinical predictors of adverse outcome were determined using univariate logistic regression.

**Findings:**

Of 724 suspected cases identified, 408 were confirmed as having encephalitis (58% infectious, 23% immune mediated, 19% of unknown aetiology). Recovery was incomplete in 58% of children at 12 months. Children with infectious encephalitis presented younger (median age 1.4 years) and showed divergent trajectories of outcome progression from discharge to 12 months. Immune mediated encephalitis affected mainly older children (median age 7.5 years) and overall showed trajectory to improved neurological outcome at 12 months relative to discharge. Hospital length of stay (OR = 1.02, 95% CI [1.01, 1.03]; p = 0.001), ICU length of stay (OR = 1.07, CI 1.01, 1.13]; p = 0.004), personality/behavioural change >24 h (OR = 1.91, 95% CI [1.01, 3.61]; p = 0.045), motor weakness (OR = 2.05, 95% CI [1.16, 3.64]; p = 0.013), and MRI consistent with encephalitis (OR = 2.22, 95% CI [1.19, 4.13]; p = 0.012), were significantly associated with worse neurological outcomes at 12 months.

**Interpretation:**

Encephalitis in children results in a considerable ongoing disease burden with clinical features and outcome differing based on disease aetiology. Neurological deficits may manifest over time and close monitoring of children for at least 12 months is warranted.

**Funding:**

National Health and Medical Research Council (NHMRC) centres for research excellence (CRE) in critical infections (APP1001021) to CJ, RB. NHMRC post-graduate scholarship (GNT1074547) and early career fellowship (GNT1145817) to PB.

**Research in context**

**Evidence before this study**

A literature search was conducted using the Medline and Embase databases to identify studies up to June 2025 which described long term neurological, and developmental outcomes in children with encephalitis. These studies were predominantly retrospective in design and often focused on a single aetiology. Previous systematic reviews, including one conducted by our research team in 2016, have demonstrated the significant ongoing disease burden for children post-encephalitis, however the small sample sizes, and methodological heterogeneity in classifying aetiology, and assessing outcomes, has made characterisation of long-term outcomes difficult. A small number of studies have proposed clinical markers of disease severity as predictive of long-term neuro-morbidity, but there is inconsistency between studies, in part because of variability in the primary neurological outcome assessed, which have included cognitive domains, functional outcomes, or quality of life.

**Added value of this study**

This multi-centre, prospective cohort study comprehensively describes the long-term neurological outcome of children aged 0 to <15 years with all cause encephalitis. It used standardised tools for the assessment of outcome, and consensus guidelines published in 2013 to classify case aetiology. Additionally, the study assessed neurological outcome at 3 months and 12 months post-discharge affording the description of trends in outcome progression by aetiology. To the best of our knowledge, this is the first study to determine statistically significant predicators of adverse long-term outcome of all-cause childhood encephalitis based on early clinical and investigation characteristics.

**Implications of all the available evidence**

Childhood encephalitis results in a considerable long-term burden of disease that has lifelong implications. Clinical severity, and progression of neurological deficits vary depending on aetiology, which itself is associated with demographic characteristics. Worse neurological outcome at 12 months post-discharge is associated with a small number of key evaluable clinical variables. Children require long-term monitoring following acute encephalitis, the intensity of which may be adjusted based on age, aetiology and other risk factors.

## Introduction

Encephalitis is inflammation of the brain parenchyma that can result in severe adverse neurological consequences or death,^1^ causing a considerable burden of disease worldwide.^2^ Clinical encephalitis results from direct brain infection, or immune processes with or without antecedent infection.^1^ In Australia, encephalitis in children can result in short-term neurological consequences or death in up to one third of cases.^3^

Due to the heterogenous causation and presentation of encephalitis, advancement in diagnosis and treatment approaches continues to be challenging.^4^ When an infectious cause is suspected, the causative agent if often not found.^5^ Furthermore, characterisation of long-term outcomes has been similarly hampered.^6^ In 2013, international guidelines including a consensus case definitions were published to assist researchers standardise case identification and investigations into encephalitis aetiology, treatment and outcomes.^4^

Previous studies describing the long-term outcomes of childhood encephalitis often focused on a single aetiology, were retrospective, had small sample sizes, and used a variety of different outcome measures. A systematic review and meta-analysis conducted by Khandaker et al.^6^ showed that long-term recovery was incomplete in nearly half of children presenting with acute infectious encephalitis. Deficits were noted across behavioural, cognitive and other developmental domains, with some children experiencing persisting motor deficits and seizures.^6^ Diagnostic approaches, follow-up period, and outcome measures varied between studies included in the review, and most studies had a retrospective design, hence misclassification and selection bias may affect the results.^6^

Herpes Simplex Virus (HSV) encephalitis has been associated with a relatively higher prevalence of long-term sequelae, especially among neonates, in who neurological deficits were not apparent at discharge.^6^, ^7^, ^8^, ^9^, ^10^ A similar pattern has been seen with encephalitis caused by parechovirus, which primarily affects neonates.^11^^,^^12^ In contrast, children with immune-mediated causes of encephalitis, such as Anti N-methyl-D-aspartate receptor (NMDAR) encephalitis and acute disseminated encephalomyelitis (ADEM), are more likely to have a good outcome, with resolution of deficits over time.^13^, ^14^, ^15^, ^16^, ^17^, ^18^ There is, however, inconsistency between studies across aetiological subgroups, with some suggesting resolution of deficits,^13^^,^^17^^,^^19^^,^^20^ while others showed long-term deficits in children who were deemed fully recovered at discharge.^7^^,^^9^^,^^11^^,^^18^^,^^21^^,^^22^

Whilst previous studies have made progress in characterising long-term morbidity of encephalitis for individual causes, little is understood about overall predictive factors for long-term neuromorbidity.^6^ The heterogeneity of methods used to classify syndromes, and lack of consistency in outcome assessment tools, are considerable limitations of the currently published research that has complicated any evaluation of specific factors predictive of adverse outcomes. Some studies have suggested that markers of disease severity such as admission to intensive care unit (ICU), seizure, low Glasgow coma scale (GCS) score, abnormal electroencephalogram (EEG), abnormal magnetic resonance imaging (MRI), duration of hospital stay and number of clinical features at presentation may be associated with adverse long-term outcomes,^14^^,^^21^, ^22^, ^23^, ^24^, ^25^, ^26^ however there is inconsistency across studies.

Therefore, the aim of this multicentre, prospective cohort study was to describe the clinical features of all-cause acute childhood encephalitis, as well as neurological sequelae at discharge, immediately post-discharge and at 12 months, using standardised methods to assess functional outcome, developmental delay, and quality of life. In this study we described post-hospitalisation outcomes by aetiology and determined risk factors for adverse long-term outcome.

## Methods

### Data collection

Data were collected as part of the Australian Childhood Encephalitis (ACE) study, a prospective cohort study coordinated through the Paediatric Active Enhanced Disease Surveillance (PAEDS) network.^27^^,^^28^ PAEDS (www.paeds.org.au) is an active, sentinel site surveillance network running in eight tertiary paediatric hospitals, where specialist trained research nurses screen health records to identify patients with suspected encephalitis.^27^^,^^28^ Nurses at each site completed case report forms for suspected cases of encephalitis using clinical records and where necessary liaison with caring clinicians, recording clinical features at presentation, patient risk factors, and outcome at discharge using the Glasgow outcome scale (GOS).^27^ Case investigations, imaging, and treatments were at the discretion of the treating physician.^28^

An expert panel, which included a paediatric infectious diseases and encephalitis specialist clinician, a specialist clinical paediatric neurologist, a paediatric infectious diseases epidemiologist, and paediatric clinical microbiologist, categorised each presentation into causal sub-groups using the international consensus case definitions, as either infectious encephalitis, immune-mediated encephalitis, encephalitis of unknown aetiology, or not encephalitis using the process described in Britton et al.^28^ The causal relationship between aetiological agent and episode was determined using criteria published by Granerod et al.^29^ Case categorisation and causal relationships were determined by consensus among all members of the expert panel, approximately 3 months post-admission. The expert panel had access to the collected data as well as original imaging reports and hospital discharge summaries as well as test results, but was not able to request additional testing beyond that undertaken by treating physicians, to further categorise cases of unknown aetiology, nor to determine a causative agent.

From March 2014 to June 2018 patients were enrolled in the Discovery of Infectious causes of Cryptic Encephalitis (DICE) outcomes study. Follow-up data were collected at two timepoints; directly following discharge and at 12 months post discharge. Follow-up was undertaken by phone interview with a parent or guardian using standardised questionnaires. Interviews not completed within three months of discharge, and 15 months, respectively were not pursued further and the child was considered as lost to follow-up for that outcome timepoint.

The primary measure used to determine neurological outcome was the Liverpool Outcome Score (LOS), which remains the only scale developed and validated specifically for measuring outcomes following childhood encephalitis.^30^, ^31^, ^32^ Using a 15-item questionnaire, the carer was asked to compare cognition, behaviour and motor functioning at follow-up relative to the child’s performance prior to their admission.^30^ Each response was given a score from 2 to 5 depending on the severity of their disability, with the lowest score across all domains representing their overall outcome.^30^ A score of 5 is considered as full recovery, scores of 4 or 3 represent minor or moderate sequelae respectively but “compatible with independent living”, and 2 indicates severe sequelae and dependency on a carer.^30^ A score of 1 was assigned if the child is deceased.^30^

Developmental outcomes were further assessed using the Ages and Stages Questionnaire, third edition (ASQ®-3)^33^ for children aged 5.5 years or younger at the time of follow-up interview. Patient health-related quality of life was assessed using the PedsQL™ questionnaire,^34^^,^^35^ and carer quality of life using the short form health survey SF-12v2.^36^

### Statistical analysis

All statistical analyses were conducted using the Stata statistical analysis software (version 19.5, StataCorp LLC, College Station, TX, USA). Alluvial plots were constructed using R (version 4.4.1, R Foundation for Statistical Computing, Vienna, Austria). Quantitative data have been presented as median values with interquartile range for continuous variables, and frequencies and proportions for categorical variables. Patient characteristics, clinical features, investigation findings and outcome scores have been compared between the three causal categories; infectious encephalitis, immune-mediated encephalitis, and encephalitis of unknown aetiology.

Potential predictive factors for worse neurological outcome according to the primary outcome (LOS), were determined using univariate ordinal logistic regression.

### Ethics approval

Ethics approval was granted by the Sydney Children’s Hospital Network Human Research Ethics Committee (HREC/18/SCHN/72 and HREC/13/SCHN/191) with local governance at each participating hospital site. Written informed consent was obtained for each child from their parent or carer.

### Role of the funding source

The funders played no role in the design of the study or analysis of data, nor did they contribute to the decision to publish.

## Results

### Patient characteristics and aetiology

**Fig. 1:**
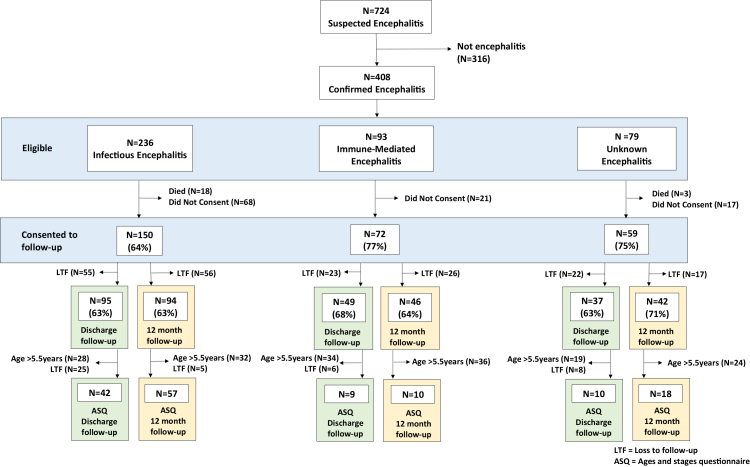
**Case flowchart for cases of suspected encephalitis from March 2014 to Jun 2018 who were eligible for study inclusion**.

The diagnostic and aetiological categorisation of patients included in the study is shown in Fig. 1. Across the five hospitals where surveillance was conducted, 724 children were identified as having suspected encephalitis between March 2014 and June 2018, of whom 408 (56%) were confirmed to be encephalitis by an expert panel.^28^ These cases were divided into three broad subgroups according to their aetiology. There were 236 (58%) cases of encephalitis with an infectious cause, 93 (23%) were immune-mediated, and 79 (19%) were of unknown aetiology. Of the infectious cases, 145 (61%) had a viral cause identified, 59 (25%) bacterial, and 29 (12%) were mixed infections with investigations detecting multiple possible causative agents. There was one case each of fungal, protozoal, and helminth infections. Of the viral causative agents, the most common were parechovirus (n = 39, 27%), and enteroviruses (n = 35, 24%). The most common causes of immune-mediated encephalitis were ADEM (n = 69, 74%), and anti-NDMAR encephalitis (n = 18, 19%).

The demographics, and medical history for the study cohort are shown in Table 1. The proportion of male patients with suspected encephalitis was 57%, with similar proportions between the diagnostic categories. The median age at presentation was 1.4 years (interquartile range [IQR], 0.1–7.5 years) for infectious encephalitis, 7.5 years (IQR, 4.6–12.3 years) for immune-mediated encephalitis, and 6.7 years (IQR, 1.7–10.4 years) for encephalitis of unknown aetiology. The proportion of children with infectious encephalitis less than one year old at presentation was 43%. A family history of autoimmune conditions (28%) was more frequent in the immune-mediated group compared to those with infectious encephalitis (14%).

**Table 1:** Cohort demographics and past medical history.

	Diagnosis category–encephalitis			Total
	Infectious	Immune-mediated	Unknown	
	(n = 236)	(n = 93)	(n = 79)	(n = 408)
Female sex	98 (42%)	43 (46%)	37 (47%)	178 (44%)
Median age, y (IQR)	1.4 (0.1–7.5)	7.5 (4.6–12.3)	6.7 (1.7–10.4)	4.0 (0.6–9.6)
Age category				
0–1 years	101 (43%)	4 (4%)	11 (14%)	116 (28%)
1–5 years	59 (25%)	23 (25%)	24 (30%)	106 (26%)
5–10 years	39 (17%)	31 (33%)	23 (29%)	93 (23%)
10–15 years	37 (16%)	35 (38%)	21 (27%)	93 (23%)
SEIFA quintile^a^				
Lowest	62 (27%)	17 (18%)	18 (23%)	97 (24%)
2nd Lowest	29 (13%)	17 (18%)	12 (15%)	58 (14%)
Middle	51 (22%)	21 (23%)	13 (17%)	85 (21%)
2nd Highest	46 (20%)	21 (23%)	16 (21%)	83 (21%)
Highest	43 (19%)	16 (17%)	19 (24%)	78 (19%)
Primary home language not English	25/151 (17%)	7/59 (12%)	8/45 (18%)	40/255 (16%)
Median birth weight, g (IQR)^b^	3150 (2770–3700)	3345 (3000–4000)	3500 (3000–3800)	3260 (2895–3770)
Median gestational age, weeks (IQR)^c^	38 (37–40)	40 (38–40)	39 (38–40)	39 (37–40)
Immunisation status^d^				
Not immunised	18 (16%)	0 (0%)	1 (3%)	19 (10%)
Partial	11 (10%)	1 (3%)	5 (14%)	17 (9%)
Up to date	83 (74%)	34 (97%)	31 (84%)	148 (80%)
FHx epilepsy	31/192 (16%)	9/82 (11%)	11/71 (15%)	51/345 (15%)
FHx MS	8/181 (4%)	4/82 (5%)	4/70 (6%)	16/333 (5%)
FHx autoimmune	26/180 (14%)	23/82 (28%)	15/70 (21%)	64/332 (19%)

Data presented as no. (%) unless otherwise indicated.

IQR = Interquartile range; SEIFA = Socio-Economic Indexes for Areas; FHx = Family history; MS = Multiple sclerosis.

a SEIFA quintiles are determined by the Australian Bureau of Statistics, ranking the socio-economic status of local areas in Australia based on national census data. The centiles presented are based on the postcode of the child’s home address.

b Birth weight data were available for 291/408 cases.

c Gestational age data were available for 140/408 cases.

d Age eligible immunisation status as reported by the parent or guardian and verified by review of the Australian Immunisation Register. Up to date may not include the seasonal influenza vaccine.

### Clinical features and severity of presentation

The overall severity of presentation across the three causal subgroups is shown in Table 2. There were 20 deaths due to encephalitis (case fatality rate, 5%); 18 due to infectious causes, and 2 of unknown cause (Table 2). The proportion of cases admitted to ICU was 65% for infectious causes, compared to 27% for immune-mediated causes, and 51% for unknown causes (Table 2). The proportion of patients with infectious encephalitis who presented with a GCS score of less than 9 was 28%, compared to 9% of patients with immune-mediated encephalitis (Table 2).

**Table 2:** Clinical features–overall severity of presentation.

	Diagnosis category–encephalitis			Total
	Infectious	Immune-mediated	Unknown	
	(n = 236)	(n = 93)	(n = 79)	(n = 408)
Median hospital stay length, days (IQR)	10 (7–23)	12 (6–30)	9 (5–17)	10 (6–14)
ICU Admission	154 (65%)	25 (27%)	40 (51%)	219 (54%)
Median ICU stay length, days (IQR)	4 (2–8)	3 (2–8)	2 (1–4)	3 (2–7)
GCS category				
13–15	64 (44%)	57 (77%)	34 (53%)	155 (55%)
9–12	40 (28%)	10 (14%)	15 (23%)	65 (23%)
3–8	41 (28%)	7 (9%)	15 (23%)	63 (22%)
Median number of signs and symptoms at presentation^a^	8 (6–11)	9 (7–11)	9 (8–11)	9 (7–11)
Deceased	18 (8%)	0 (0%)	2 (3%)	20 (5%)

Data presented as no. (%) unless otherwise indicated.

IQR = Interquartile range; ICU = Intensive Care Unit; GCS = Glasgow Coma Scale.

a Signs and symptoms list: altered level of consciousness/lethargy, decreased response to the environment, decreased eye contact, decreased arousability, seizure with loss of consciousness, personality/behaviour change, fever, focal seizure, cranial nerve abnormality, visual impairment, motor weakness, altered deep tendon reflexes, cerebellar dysfunction, abnormal movements, bladder/bowel dysfunction, irritability, headache, cough/coryza, diarrhoea, vomiting, rash.

Signs and symptoms at presentation across the three subgroups of encephalitis are presented in Supplementary Table S1. Several differences between the groups were evident. Both focal seizure, and seizure with loss of consciousness were more than twice as likely to occur in children with infectious or unknown aetiology, compared to those with immune-mediated encephalitis. On the other hand, focal neurological deficits such as cranial nerve abnormality, visual impairment, motor weakness, cerebellar dysfunction, and bladder/bowel dysfunction were more predominant in the immune-mediated encephalitis sub-group. Constitutional and global neurological symptoms, other than seizure, were largely similar between groups.

Supplementary Table S2 shows the results of key diagnostic investigations. MRI consistent with encephalitis, and pleocytosis were more common in immune-mediated encephalitis compared infectious and unknown cause.

The pattern of signs, symptoms, and investigation results suggest a similarity between the infectious encephalitis, and encephalitis of unknown cause, that may warrant further exploration.

### Discharge and long-term outcomes

Of the 408 cases of confirmed encephalitis, 281 (69%) consented to follow-up. Outcome data were collected for 182 patients following discharge (65%), and 182 (65%) patients at 12 months (Fig. 1). The main reason for loss to follow-up was that the parent or guardian could not be contacted within the time-period specified.

Table 3 describes neurological outcome using the GOS assessed prior to discharge, as well as the primary outcome measure, the LOS,^31^ at discharge and at 12 months, for each of the subcategories of encephalitis.

**Table 3:** Neurological outcomes following acute encephalitis at discharge and 12 months.

	Diagnosis category–encephalitis			Total
	Infectious	Immune-mediated	Unknown	
GOS score at discharge^a^				
Death	18 (8%)	0 (0%)	3 (4%)	21 (5%)
Vegetative state	3 (1%)	0 (0%)	0 (0%)	3 (1%)
Severe disability	12 (5%)	4 (4%)	2 (3%)	18 (4%)
Moderate disability	49 (21%)	28 (30%)	13 (16%)	90 (22%)
Good recovery	152 (65%)	60 (65%)	61 (77%)	273 (67%)
LOS lowest score at discharge^b^				
Severe sequelae	11 (12%)	7 (15%)	2 (6%)	20 (11%)
Moderate sequelae	23 (25%)	15 (31%)	12 (33%)	50 (28%)
Minor sequelae	24 (26%)	14 (29%)	10 (28%)	48 (27%)
Full recovery	35 (38%)	12 (25%)	12 (33%)	59 (33%)
LOS lowest score at 12 months^c^				
Severe sequelae	17 (19%)	7 (16%)	5 (12%)	29 (16%)
Moderate sequelae	18 (20%)	9 (20%)	10 (24%)	37 (21%)
Minor sequelae	22 (24%)	8 (18%)	8 (20%)	38 (21%)
Full recovery	34 (37%)	21 (47%)	18 (44%)	73 (41%)

Data presented as no. (%). The denominator for LOS lowest score varies, and for simplicity of presentation, has not been included.

GOS = Glasgow Outcome Scale; LOS = Liverpool Outcome Score.

a Assessed at discharge. Two cases of infectious encephalitis, and one case of immune-mediated encephalitis did not have GOS score recorded at discharge.

b Assessed within 3 months of discharge. Of the 181 cases followed up within 3 months, four cases (two infectious, one immune-mediated and one unknown) had incomplete interview data and LOS lowest score was not calculated for these cases.

c Assessed between 12 months and 15 months of discharge. Of the 182 cases followed up at 12 months, five cases (three infectious, one immune-mediated and one unknown) had incomplete interview data and LOS lowest score was not calculated for these cases.

Full recovery at 12 months was achieved in 41% of patients with all-cause encephalitis according to the LOS^31^ (Table 3). The proportion of children with infectious encephalitis with full recovery was 38% at discharge and remained at a similar proportion (37%) at 12 months (Table 3). Immune-mediated encephalitis had a 25% recovery rate at discharge but improved to 47% at 12 months (Table 3). Neurological outcome at 12 months according to LOS stratified by aetiology is shown in Table 4. For children with encephalitis due to parechovirus, 7 of 15 children for whom data were available showed severe neurological sequelae at 12 months (Table 4). HSV encephalitis also showed worse outcomes than average, with 8 out of 11 children having moderate or severe sequelae at 12 months (Table 4).

**Table 4:** Neurological Outcome at 12 months by aetiology.

	LOS lowest score at 12 months^a^				
	Severe sequelae	Moderate sequelae	Minor sequelae	Full recovery	Total
N	29 (16%)	37 (21%)	38 (21%)	73 (41%)	177
Encephalitis causal groups					
Influenza	2 (20%)	2 (20%)	4 (40%)	2 (20%)	10
HSV	3 (27%)	5 (45%)	0 (0%)	3 (27%)	11
Parechovirus	7 (47%)	1 (7%)	3 (20%)	4 (27%)	15
Enterovirus	2 (11%)	4 (21%)	2 (11%)	11 (58%)	19
Mycoplasma	1 (11%)	1 (11%)	5 (56%)	2 (22%)	9
Bacterial meningoencephalitis	1 (9%)	3 (27%)	1 (9%)	6 (55%)	11
Other infectious^b^	1 (6%)	2 (13%)	7 (44%)	6 (38%)	16
ADEM	6 (17%)	6 (17%)	8 (22%)	16 (44%)	36
Other autoimmune^c^	1 (11%)	3 (33%)	0 (0%)	5 (56%)	9
Unknown	5 (12%)	10 (24%)	8 (20%)	18 (44%)	41

Data presented as no. (%).

LOS = Liverpool Outcome Score.

a Assessed between 12 months and 15 months of discharge. Five cases had incomplete interview data and LOS lowest score was not able to be calculated.

b Includes: Mixed or single aetiology including infective causes listed above and/or Adenovirus, Cryptococcus, Epstein Barr Virus, Human Herpesvirus 6, Middle Eastern Respiratory Syndrome Coronavirus, Norovirus, Parainfluenza Virus, Respiratory Syncytial Virus, Rotavirus.

c Includes: Anti-NMDAR (6), immune-mediated with unknown causative agent (2), anti-GAD (1).

### Progression of outcome from discharge to 12 months

**Fig. 2:**
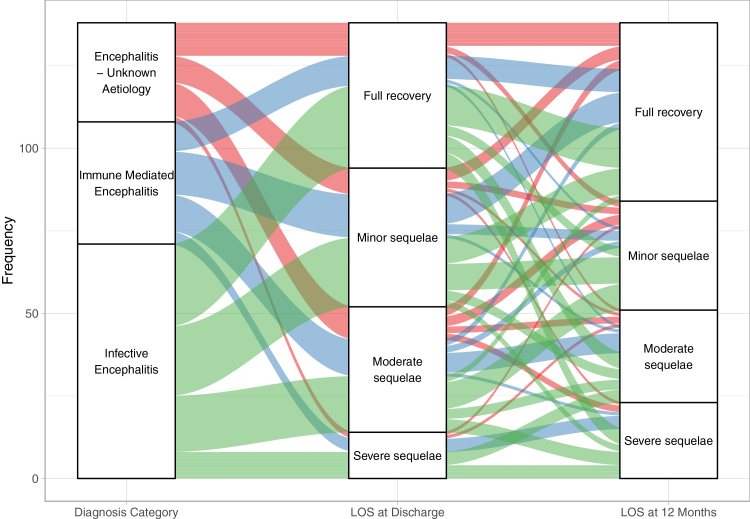
**Longitudinal changes in neurological outcome between discharge and 12 months by diagnosis category.** The case flows for infective, immune-mediated and unknown aetiology are shown in green, blue and red respectively. There are three nodes, the first divided into the three aetiological subgroups, and the second and third divided into the LOS severity category at discharge and at 12 months respectively. The height of the strata at the second and third node represents the number of cases in each outcome severity category, and the height of the flow at each node, represent the number of cases from each aetiology. LOS = Liverpool Outcome Scor

Fig. 2 presents an alluvial chart showing the change in the LOS from discharge to 12-month follow-up divided by diagnosis category. There were no cases with severe sequelae at discharge that fully recovered at 12 months follow-up (Fig. 2). Although Table 3 shows better outcomes for immune-mediated encephalitis at 12 months, the improvement was mostly in a single category, with 2 of 11 cases with moderate sequelae at discharge improving to fully recovered (Fig. 2). When progression of outcome was stratified by age and causative agent, younger children with infectious encephalitis, especially those aged less than 1 year at presentation, showed a divergence in the trajectory of outcome progression from discharge to 12 months (Supplementary Fig. S1 through Supplementary Fig. S4). Of the 25 cases of infectious encephalitis which showed full recovery at discharge, 13 (52%) showed neurological deficits at 12 months (Fig. 2).

Developmental outcome according to the Ages and Stages Questionnaire (ASQ®-3)^33^ at discharge and 12 month follow-up are presented in Supplementary Table S3 and Supplementary Table S4 respectively. Patients with infectious encephalitis showed typical gross motor and fine motor development in 59% and 62% of cases at discharge (Supplementary Table S3), and 56% and 64% of cases at 12 months follow-up (Supplementary Table S4). Patients with immune-mediated encephalitis showed typical gross motor and fine motor development in 78% and 44% of cases at discharge (Supplementary Table S3), improving to 100% and 90% of cases at 12 months follow-up (Supplementary Table S4). Personal and social development for immune-mediated encephalitis improved from 56% of cases at discharge (Supplementary Table S3) to 90% of cases at 12-month follow-up (Supplementary Table S4).

Children’s health related quality of life, as measured by the PedsQL™ score,^34^ and carer quality of life according the short form health survey SF-12v2^36^ are shown in Supplementary Table S5.

### Predictors of adverse outcome

The association between patient characteristics and clinical features at presentation, and LOS at 12 months were assessed using univariate ordinal regression (Supplementary Appendix D) with variables with a p value of less than 0.20 shown in Table 5. Some of the variables shown in Supplementary Appendix D with p < 0.20 were excluded due to low sample size or lack of clinical relevance. There were several clinical factors associated with worse outcome at 12 months using the LOS^31^ as shown in Table 5. Hospital length of stay (odds ratio (OR) = 1.02, 95% CI [1.01, 1.03]; p = 0.001), ICU length of stay (OR = 1.07, CI 1.01, 1.13]; p = 0.004), personality/behavioural change >24 h (OR = 1.91, 95% CI [1.01, 3.61]; p = 0.045), motor weakness (OR = 2.05, 95% CI [1.16, 3.64]; p = 0.013), and MRI consistent with encephalitis (OR = 2.22, 95% CI [1.19, 4.13]; p = 0.012), were all significantly associated (p < 0.05) with worse neurological outcome (Table 5). The univariate analysis for all variables in shown in Supplementary Appendix D.

**Table 5:** Predictive factors for worse neurological outcome at 12 months.

Predictive factor	Univariate analysis		
	n	OR (95% CI)	p value
Patient characteristics			
Age (years)	177	0.96 (0.9–1.01)	0.118
Clinical severity			
Hospital length of stay length (days)	177	1.02 (1.01–1.03)	0.001
ICU Admission	177	1.53 (0.89–2.62)	0.124
ICU length of stay (days)	84	1.07 (1.01–1.13)	0.004
Inotropes administered	177	2.80 (1.03–7.62)	0.044
GCS	127	0.92 (0.84–1.01)	0.083
Number of signs and symptoms at presentation^a^	177	1.09 (1–1.19)	0.056
Clinical signs			
Decreased response to environment	161	1.51 (0.85–2.67)	0.156
Decreased eye contact	150	1.59 (0.88–2.86)	0.120
Focal seizure	161	1.62 (0.91–2.87)	0.101
Seizure with LOC	170	1.66 (0.95–2.88)	0.073
Personality/behaviour change^b^	174	1.91 (1.01–3.61)	0.045
Motor weakness	163	2.05 (1.16–3.64)	0.013
Abnormal movements	169	2.09 (1.19–3.66)	0.010
Bladder/bowel dysfunction	160	1.72 (0.94–3.16)	0.079
Investigations			
MRI consistent with encephalitis	167	2.22 (1.19–4.13)	0.012

p values calculated using ordinal logistic regression.

OR = Odds ratio; ICU = Intensive care unit; GCS = Glasgow coma scale; LOC = Loss of consciousness; MRI = Magnetic resonance imaging.

a Signs and symptoms list: altered level of consciousness/lethargy, decreased response to the environment, decreased eye contact, decreased arousability, seizure with loss of consciousness, personality/behaviour change, fever, focal seizure, cranial nerve abnormality, visual impairment, motor weakness, altered deep tendon reflexes, cerebellar dysfunction, abnormal movements, bladder/bowel dysfunction, irritability, headache, cough/coryza, diarrhoea, vomiting, rash.

b Lasting greater than 24 h.

## Discussion

Here, we have comprehensively reported long-term (12-month) outcomes of all-cause encephalitis in children from a unique, national, prospective cohort from the Southern hemisphere. Our findings confirm the considerable long term neurological burden of encephalitis in children. This study showed that patient characteristics, features of clinical presentation, and neurological outcomes differed between aetiological subgroups. The longitudinal trajectory of sequelae also differed, with the proportion of children with complete recovery following immune-mediated encephalitis almost doubling between discharge and 12-months follow-up. In contrast the proportion of children showing full recovery from infectious encephalitis was unchanged. Despite the heterogeneity of disease presentation and progression of neurological outcome, our analysis identified a small number of evaluable clinical predictors of worse neurological outcome at 12 months that showed robust statistical significance.

### Disease severity and progression of outcomes

Strikingly, approximately 1 in 6 children with all cause encephalitis showed severe neurological disability resulting in ongoing dependency for activities of daily living on a carer. The rate of severe sequelae amongst children with an infectious cause (19%) is significantly higher than the previously reported pooled prevalence of 6.7% (95% CI [4.5%, 8.8%]) for infectious encephalitis in a systematic review by Khandaker et al.^6^ This is in addition to acute fatality in 1 in 18 children (case fatality rate of 6%) also considerably higher than the pooled rate of 2.7% (95% CI [1.7%, 4.5%]) previously reported.^6^ A further 2 in 5 children had incomplete recovery at 12 months, hence the prevalence of death or disability from childhood encephalitis was greater than 60%. This long-term neurological burden needs greater attention and is more substantial than that of neonatal stroke,^37^ and similar to that of traumatic brain injury in children.^38^

Children with infectious encephalitis were younger, with almost half aged less than 1 year at disease onset. They were more likely to present with more severe disease, and two thirds of patients were admitted to ICU. As the age of children with infectious encephalitis was much younger, neurological symptoms and signs at presentation may be underreported or less reliably assessed. A considerable proportion of cases of infectious encephalitis showed worse outcome at 12 months than was evident at discharge, with variation in progression of outcome, especially in children aged less than 1 for whom parechovirus and enterovirus were the predominant causative agents. Parechovirus and HSV encephalitis showed worse neurological outcome than other causative agents. This pattern of neurological outcome progression is consistent with previous studies of parechovirus central nervous system (CNS) infection in infants,^11^ as well as HSV encephalitis.^6^ Careful monitoring is warranted for these young children for at least 12 months, for whom the severity of the sequelae from their illness may manifest over time.

Children with immune-mediated encephalitis were older, and more likely to present with focal neurology than those with infectious encephalitis. Children with immune-mediated encephalitis were less frequently admitted to ICU, no cases resulted in death, and neurological outcome improved over 12 months. This is consistent with previous studies which showed “good outcome” in over 80% of cases.^13^, ^14^, ^15^ Two of these studies^13^^,^^14^ used the modified Rankin Scale (mRS)^39^ to measure neurological outcome however, they are comparable in assessing level of disability. Developmental outcomes for children with immune-mediated encephalitis measured using the ASQ®-3 questionnaire^33^ showed similar improvements over time with 90% or greater showing typical development at 12 months, and no patients in the lowest category of requiring further assessment. These findings are similar to previously published studies,^13^^,^^17^^,^^19^ however they should be treated with caution due to the small sample size (n = 9 at discharge and n = 10 at 12-month follow-up).

Although immune-mediated encephalitis generally shows good long-term recovery, several studies have reported higher rates of residual neurological deficits than observed in our cohort. In a multicentre retrospective cohort study of anti-NMDAR encephalitis conducted recently in France, cognitive impairment was present in 45% of children, and 70% reported academic difficulties at a median follow-up of 40 months (range, 25–53 months); notably, 92% required rehabilitation.^14^ A smaller single centre study in Israel also showed increased rates of attention deficit hyperactivity disorder (ADHD), and low intelligence quotient (IQ) in children at a mean follow-up of 5.5 years (range, 1–14.5 years).^18^ These more subtle cognitive and neuropsychological sequelae may be underestimated in studies relying on crude outcome measures which primarily assess functional status. Clinicians and caregivers should therefore remain alert to potential behavioural or academic difficulties and consider further neuropsychological assessment at timepoints such as the start of primary or secondary school, or when any concerns arise.

This study identified hospital length of stay, ICU length of stay, personality/behavioural change, motor weakness, and MRI consistent with encephalitis as significantly associated with worse neurological outcome at 12 months for all-cause encephalitis. These factors are similar to a recently published prospective multi-centre study of infectious encephalitis in adults conducted in France which showed abnormal brain imaging and ICU admission to be significantly associated with neurological outcome at 12 months.^40^

This constellation of clinical factors associated with worse neurological outcome at 12 months suggests more diffuse neurological impairment at the time of presentation is associated with a higher burden of disease in the long-term, however larger scale studies will be needed to validate the utility of these variables as predictive indices for long-term neurological morbidity following acute childhood encephalitis.

### Strengths and limitations

The strengths of this study are its prospective study design, and case identification through an active surveillance program (PAEDS network^28^) which eliminated selection bias and ensured comprehensive collection of demographic and clinical data at presentation. The study considers all-cause encephalitis, allowing comparisons of clinical features at presentation and neurological sequelae between the different aetiologies of encephalitis, using standardised methods of data collection and outcome measurement.

A limitation of this study was the proportion of cases who did not consent to follow-up, and those lost to follow-up. Of the 408 cases of confirmed encephalitis, follow-up data was available for 182 at 12 months (Fig. 1) reducing both the power of our analysis, and generalisability of findings due to possible selection bias. This is especially significant in relation to determining developmental outcomes for children up to 5.5 years of age, where 85 children had data available at 12-month follow-up (Fig. 1). An overall comparison of the demographic, clinical, and outcome at discharge data for patients who completed 12-month follow-up, and those lost to follow-up, did not reveal any substantial imbalances amongst the measured variables, or across key causal sub-groups (Supplementary Appendix C), nevertheless follow-up selection bias cannot be discounted. Despite the loss of power and possible presence of follow-up selection bias, this is the largest of study of its kind to date to determine associations between clinical variables and adverse outcome for children hospitalised with all-cause encephalitis.

Importantly, we acknowledge there have been significant advancements in both auto-antibody testing and classification of immune mediated encephalitis since the conception of this study.^41^^,^^42^ In a recently published prospective multi-centre study in Spain, approximately half of children with immune mediated encephalitis had ADEM, a lower proportion than in this cohort, with one in three having anti-NMDAR encephalitis, and a proportion of cases with other auto-immune aetiologies not frequently tested in our study.^43^ This includes myelin oligodendrocyte glycoprotein antibody-associated disease (MOGAD),^44^ a proportion of which is ADEM-associated but also with other forms of encephalitis. It is possible that some immune mediated cases in our study may have been classified as unknown, and this may have impacted the strength of association between aetiological subgroup and neurological outcome at 12 months in our analysis.

Australia is a high-income country with associated high resource healthcare system, hence generalisability to low and middle-income countries is limited, although adverse outcomes are unlikely to be of lower prevalence in these locations.^45^ In addition, vector-borne causes of encephalitis (e.g., flaviviruses, alphaviruses) were very infrequent in our cohort, however we note the epidemiology of such infections has subsequently changed in Australia.^46^

There is no unaffected population control group for this study, hence any contribution of additional post-illness factors on encephalitis outcome, cannot be determined. Furthermore, children were only followed up for 12 months after discharge from hospital, hence the study may not have captured the full extent to which children may have subsequently recovered or not. There are studies suggesting that the prognosis of children with immune-mediated encephalitis continues to improve past 12 months^15^^,^^17^^,^^47^ however the largest improvement in outcome likely occurs in the first 12 months.^46^

### Conclusions

This is one of the most comprehensive studies published to date describing the long-term outcomes of all-cause encephalitis in children. Encephalitis results in incomplete long-term recovery in a majority of children who are admitted to hospital. The extent of neurological sequelae may not be fully evident at discharge, especially in young children who most often presented with infectious causes. Ongoing close monitoring of children post-encephalitis and early intervention is essential to mitigate impacts of this long-term burden of disease.

## Contributors

RD, RBo, CJ and PB conceptualised the study. Funding was acquired by PB, CJ, and RBo. RD, RBo, CJ, and PB developed the methodology. CB, JB, JC, NC, and HM provided resources and supervision. RBu, KP and GR curated the data. GR performed the formal statistical analysis with input and validation from LFK. GR prepared the visualizations and compiled the original draft with PB. All authors critically reviewed and edited the manuscript and approved the final version. GR and PB accessed and verified the data. GR and PB were responsible for the decision to submit the manuscript.

The Paediatric Active Enhanced Disease Surveillance (PAEDS) Network members apart from the named authors include its surveillance nurse team who identified cases, collected data and sought consent for follow-up (Emma Carey, Alissa McMinn, Kate Dohle, Anna Wylie, Karen Bellamy, Guillian Hunter, Madison Bellamy, Christine Heath, Natascha D’Angelo, Carolyn Finucane, Laura Francis, Sonia Dougherty, Natasha Doran, Sara Cook, Nicole Kerly, Elizabeth Foat, Sophie Kasz, Jocelynne McRae Nicole Dinsmore, Kathryn Meredith, Laura Rost, Shirley Wong) and its co-investigators who provided supervision and contributed to network governance across the study period (A/Prof Joshua Francis, Dr Jeremy Carr, Prof. Peter Richmond, Prof. Kristine Macartney, Prof. Nicholas Wood and Dr Anne Kynaston).

## Data sharing statement

Data from the Australian Childhood Encephalitis (ACE) cohort study is not publicly available. To request access to the data, please contact the corresponding author.

## Declaration of interests

Prof Dale was a member of a Data Safety and Monitoring Board for a Roche sponsored trial for paediatric multiple sclerosis. Prof Marshall is an investigator on clinical trials sponsored by Sanofi, Pfizer and Seqirus, but receives no personal payments from industry sponsors. Prof Marshall was a member of a Data Safety and Monitoring Board for a Glaxo-Smith-Kline sponsored trial and has received speaker travel support from GSK. All other authors have no competing interests to declare.
