# Supplementary material for: Long-term outcomes of childhood encephalitis in Australia: burden and predictors from the Australian childhood encephalitis (ACE) prospective multicentre cohort
Source: Lancet Reg Health West Pac. 2026 Aug 8;73:101951. doi: 10.1016/j.lanwpc.2026.101951 (PMC13476763; doi:10.1016/j.lanwpc.2026.101951)
Supplement: Supplementary file 1 [file mmc1.docx]

## Long-term outcomes of childhood encephalitis in Australia: Supplementary Appendices

[Appendix A: Supplementary Tables	2](#_Toc234770610)

[Appendix B: Supplementary Figures - longitudinal progression of outcomes	6](#_Toc234770611)

[Appendix C: Comparative review of cases with no follow-up and those with missing data	14](#_Toc234770612)

[Appendix D: Univariate ordinal regression	18](#_Toc234770613)

Appendix A: Supplementary Tables

[Supplementary Table 1: Clinical Features - Signs and symptoms	3](#_Toc234770614)

[Supplementary Table 2: Clinical Features - Investigations	3](#_Toc234770615)

[Supplementary Table 3: Developmental outcomes at discharge (age ≤ 5.5 years)	4](#_Toc234770616)

[Supplementary Table 4: Developmental outcomes at 12 months (age ≤ 5.5 years)	4](#_Toc234770617)

[Supplementary Table 5: Child quality of life at 12 months	5](#_Toc234770618)

[Supplementary Table 6: Carer quality of life at 12 months	5](#_Toc234770619)

**References**

1. Varni JW, Seid M, Kurtin PS. PedsQL^TM^ 4.0: Reliability and validity of the Pediatric Quality of Life Inventory^TM^ Version 4.0 generic core scales in healthy and patient populations: *Medical Care* 2001; **39**: 800–812.
2. Ware JE, Kosinski M, Turner-Bowker DM, et al. How to score version 2 of the SF-12 health survey (with a supplement documenting version 1). Lincoln, R.I.: QualityMetric Inc. ; Boston, 2002.

Supplementary Table 1: Clinical Features - Signs and symptoms

|  | **Diagnosis Category – Encephalitis** | | |  |
| --- | --- | --- | --- | --- |
|  | **Infectious** | **Immune-mediated** | **Unknown** | **Total** |
|  | **(n=236)** | **(n=93)** | **(n=79)** | **(n=408)** |
| Altered LOC/Lethargy | 213/233 (91%) | 82/92 (89%) | 74/78 (95%) | 369/403 (92%) |
| Decreased Response to Environment | 102/207 (49%) | 41/89 (46%) | 40/73 (55%) | 183/369 (50%) |
| Decreased Eye Contact | 97/190 (51%) | 39/85 (46%) | 39/75 (52%) | 175/350 (50%) |
| Decreased Arousability | 147/222 (66%) | 55/91 (60%) | 52/78 (67%) | 254/391 (65%) |
| Focal Seizure | 111/213 (52%) | 16/87 (18%) | 32/78 (41%) | 159/378 (42%) |
| Seizure with loss of consciousness | 128/224 (57%) | 18/89 (20%) | 39/78 (50%) | 185/391 (47%) |
| Personality/ Behaviour Change^a^ | 171/227 (75%) | 71/92 (77%) | 55/75 (73%) | 297/394 (75%) |
| CN Abnormality | 16/174 (9%) | 29/85 (34%) | 13/72 (18%) | 58/331 (18%) |
| Visual Impairment | 32/165 (19%) | 25/83 (30%) | 9/67 (13%) | 66/315 (21%) |
| Motor Weakness | 77/196 (39%) | 52/89 (58%) | 28/74 (38%) | 157/359 (44%) |
| Altered DTR | 47/182 (26%) | 29/80 (36%) | 16/70 (23%) | 92/332 (28%) |
| Cerebellar Dysfunction | 61/174 (35%) | 54/85 (64%) | 33/71 (46%) | 148/330 (45%) |
| Abnormal Movements | 101/219 (46%) | 37/91 (41%) | 39/76 (51%) | 177/386 (46%) |
| Bladder/Bowel Dysfunction | 40/191 (21%) | 45/90 (50%) | 15/72 (21%) | 100/353 (28%) |
| Fever >38C | 181/230 (79%) | 58/90 (64%) | 57/77 (74%) | 296/397 (75%) |
| Headache | 55/127 (43%) | 60/83 (72%) | 37/64 (58%) | 152/274 (55%) |
| Irritability | 143/219 (65%) | 55/91 (60%) | 44/78 (56%) | 242/388 (62%) |
| Cough/Coryza | 49/110 (45%) | 15/35 (43%) | 16/37 (43%) | 80/182 (44%) |
| Diarrhoea | 61/226 (27%) | 8/93 (9%) | 16/79 (20%) | 85/398 (21%) |
| Vomiting | 104/230 (45%) | 52/92 (57%) | 37/78 (47%) | 193/400 (48%) |
| Rash | 69/232 (30%) | 14/93 (15%) | 15/79 (19%) | 98/404 (24%) |
| Data presented as no. (%) unless otherwise indicated | | | | |
| Abbreviations: LOC = Level of consciousness; CN = Cranial Nerve; DTR = Deep tendon reflexes | | | | |
| ^a^Lasting greater than 24 hours | | | | |

Supplementary Table 2: Clinical Features - Investigations

|  | **Diagnosis Category – Encephalitis** | | |  |
| --- | --- | --- | --- | --- |
|  | **Infectious** | **Immune-mediated** | **Unknown** | **Total** |
|  | **(n=236)** | **(n=93)** | **(n=79)** | **(n=408)** |
| MRI consistent with encephalitis |  |  |  |  |
| No | 61 (28%) | 16 (17%) | 27 (36%) | 104 (27%) |
| Yes | 145 (67%) | 75 (81%) | 36 (48%) | 256 (67%) |
| Inconclusive | 9 (4%) | 2 (2%) | 12 (16%) | 23 (6%) |
| CT Results |  |  |  |  |
| Normal | 54 (49%) | 27 (64%) | 34 (77%) | 115 (58%) |
| Abnormal | 51 (46%) | 13 (31%) | 10 (23%) | 74 (38%) |
| Inconclusive | 6 (5%) | 2 (5%) | 0 (0%) | 8 (4%) |
| EEG Results |  |  |  |  |
| Normal | 14 (10%) | 7 (18%) | 9 (16%) | 30 (13%) |
| Abnormal | 126 (88%) | 30 (79%) | 45 (82%) | 201 (85%) |
| Inconclusive | 4 (3%) | 1 (3%) | 1 (2%) | 6 (3%) |
| CSF turbidity | 17/195 (9%) | 2/82 (2%) | 2/74 (3%) | 21/351 (6%) |
| CSF pleocytosis^a^ | 134/212 (63%) | 71/89 (80%) | 49/77 (64%) | 254/378 (67%) |
| Oligoclonal bands |  |  |  |  |
| Negative | 30 (64%) | 36 (55%) | 28 (74%) | 94 (63%) |
| Positive | 17 (36%) | 25 (38%) | 8 (21%) | 50 (33%) |
| Inconclusive | 0 (0%) | 4 (6%) | 2 (5%) | 6 (4%) |
| Data presented as no. (%) unless otherwise indicated | | | | |
| Abbreviations: MRI = Magnetic resonance imaging; CT = Computed tomography; EEG = Electroencephalogram; CSF = Cerebrospinal fluid; WBC = White blood cells | | | | |
| ^a^Defined as >15 WBC/mm³ if < 2 months old; >5 WBC/mm³ if ≥2 months | | | | |

Supplementary Table 3: Developmental outcomes at discharge (age ≤ 5.5 years)

|  | **Diagnosis Category - Encephalitis** | | | |  |
| --- | --- | --- | --- | --- | --- |
|  | **Infectious** | **Immune-mediated** | **Unknown** | **Total** | |
|  | **(n=67)** | **(n=15)** | **(n=18)** | **(n=100)** | |
| **ASQ Domains** |  |  |  |  | |
| Communication |  |  |  |  | |
| Needs Further Assessment | 7 (18%) | 0 (0%) | 1 (10%) | 8 (14%) | |
| Monitor | 3 (8%) | 4 (44%) | 1 (10%) | 8 (14%) | |
| Typical Development | 28 (74%) | 5 (56%) | 8 (80%) | 41 (72%) | |
| Gross Motor |  |  |  |  | |
| Needs Further Assessment | 11 (27%) | 2 (22%) | 2 (22%) | 15 (25%) | |
| Monitor | 6 (15%) | 0 (0%) | 1 (11%) | 7 (12%) | |
| Typical Development | 24 (59%) | 7 (78%) | 6 (67%) | 37 (63%) | |
| Fine Motor |  |  |  |  | |
| Needs Further Assessment | 9 (24%) | 3 (33%) | 1 (14%) | 13 (25%) | |
| Monitor | 5 (14%) | 2 (22%) | 0 (0%) | 7 (13%) | |
| Typical Development | 23 (62%) | 4 (44%) | 6 (86%) | 33 (62%) | |
| Problem Solving |  |  |  |  | |
| Needs Further Assessment | 5 (14%) | 0 (0%) | 1 (14%) | 6 (12%) | |
| Monitor | 4 (11%) | 0 (0%) | 0 (0%) | 4 (8%) | |
| Typical Development | 26 (74%) | 9 (100%) | 6 (86%) | 41 (80%) | |
| Personal/Social |  |  |  |  | |
| Needs Further Assessment | 11 (27%) | 2 (22%) | 1 (11%) | 14 (24%) | |
| Monitor | 8 (20%) | 2 (22%) | 3 (33%) | 13 (22%) | |
| Typical Development | 22 (54%) | 5 (56%) | 5 (56%) | 32 (54%) | |
| Data presented as no. (%) unless otherwise indicated | | | | | |
| Abbreviations: ASQ = Ages and Stages Questionnaire | | | | | |

Supplementary Table 4: Developmental outcomes at 12 months (age ≤ 5.5 years)

|  | **Diagnosis Category - Encephalitis** | | |  |
| --- | --- | --- | --- | --- |
|  | **Infectious** | **Immune-mediated** | **Unknown** | **Total** |
|  | **(n=62)** | **(n=10)** | **(n=18)** | **(n=90)** |
| **ASQ Domains** |  |  |  |  |
| Communication |  |  |  |  |
| Needs Further Assessment | 11 (20%) | 0 (0%) | 5 (29%) | 16 (19%) |
| Monitor | 14 (25%) | 0 (0%) | 1 (6%) | 15 (18%) |
| Typical Development | 31 (55%) | 10 (100%) | 11 (65%) | 52 (63%) |
| Gross Motor |  |  |  |  |
| Needs Further Assessment | 20 (36%) | 0 (0%) | 3 (18%) | 23 (28%) |
| Monitor | 4 (7%) | 1 (10%) | 2 (12%) | 7 (9%) |
| Typical Development | 31 (56%) | 9 (90%) | 12 (71%) | 52 (63%) |
| Fine Motor |  |  |  |  |
| Needs Further Assessment | 9 (17%) | 0 (0%) | 2 (13%) | 11 (14%) |
| Monitor | 10 (19%) | 0 (0%) | 5 (33%) | 15 (19%) |
| Typical Development | 34 (64%) | 9 (100%) | 8 (53%) | 51 (66%) |
| Problem Solving |  |  |  |  |
| Needs Further Assessment | 13 (25%) | 0 (0%) | 3 (19%) | 16 (21%) |
| Monitor | 7 (14%) | 1 (10%) | 2 (12%) | 10 (13%) |
| Typical Development | 31 (61%) | 9 (90%) | 11 (69%) | 51 (66%) |
| Personal/Social |  |  |  |  |
| Needs Further Assessment | 13 (23%) | 0 (0%) | 2 (12%) | 15 (18%) |
| Monitor | 8 (14%) | 1 (10%) | 2 (12%) | 11 (13%) |
| Typical Development | 36 (63%) | 9 (90%) | 13 (76%) | 58 (69%) |
| Data presented as no. (%) unless otherwise indicated | | | | |
| Abbreviations: ASQ = Ages and stages questionnaire | | | | |

Supplementary Table 5: Child quality of life at 12 months

|  | **Diagnosis Category - Encephalitis** | | |  |
| --- | --- | --- | --- | --- |
|  | **Infectious** | **Immune-mediated** | **Unknown** | **Total** |
|  | **(n=79)** | **(n=45)** | **(n=41)** | **(n=165)** |
| PedsQL Total Score^a^ | 81·0 (18·9) | 83·2 (17·1) | 83·2 (19·3) | 82·1 (18·5) |
| PedsQL Physical Score^b^ | 85·5 (18·3) | 86·9 (16·2) | 87·0 (21·6) | 86·2 (18·5) |
| PedsQL Psychological Score^c^ | 76·6 (21·2) | 80·4 (20·5) | 81·2 (19·6) | 78·7 (20·6) |
| Data presented as mean (standard deviation) | | | | |
| Abbreviations: PedsQL = Paediatric quality of life questionnaire | | | | |
| ^a^Mean = 87·6, standard deviation = 12·3 for healthy children^1^ | | | | |
| ^b^Mean = 89·3, standard deviation = 16·4 for healthy children^1^ | | | | |
| ^c^Mean = 86·6, standard deviation = 12·8 for healthy children^1^ | | | | |

Supplementary Table 6: Carer quality of life at 12 months

|  | **Diagnosis Category - Encephalitis** | | |  |
| --- | --- | --- | --- | --- |
|  | **Infectious** | **Immune-mediated** | **Unknown** | **Total** |
|  | **(n=92)** | **(n=41)** | **(n=41)** | **(n=174)** |
| SF Physical Score^a^ | 54·1 (6·0) | 53·3 (8·1) | 55·9 (4·6) | 54·3 (6·3) |
| SF Mental Score^a^ | 51·0 (8·6) | 51·5 (10·4) | 52·0 (9·6) | 51·4 (9·2) |
| Data presented as mean (standard deviation) | | | | |
| SF = Short form health survey | | | | |
| ^a^Mean = 50·0, standard deviation = 10·0 for US population^2^ | | | | |

Appendix B: Supplementary Figures - longitudinal progression of outcomes

[Supplementary Figure 1: Change in neurological outcome between discharge and 12 months by diagnosis category - Ages 0 - 1 years	7](#_Toc234770620)

[Supplementary Figure 2: Change in neurological outcome between discharge and 12 months by diagnosis category - Ages 1 - 5 years	8](#_Toc234770621)

[Supplementary Figure 3: Change in neurological outcome between discharge and 12 months by diagnosis category - Ages 5 - 10 years	9](#_Toc234770622)

[Supplementary Figure 4: Change in neurological outcome between discharge and 12 months by diagnosis category - Ages 10 to <15 years	10](#_Toc234770623)

[Supplementary Figure 5: Change in neurological outcome between discharge and 12 months by diagnosis category - Infectious encephalitis	11](#_Toc234770624)

[Supplementary Figure 6: Change in neurological outcome between discharge and 12 months by diagnosis category - Immune-mediated encephalitis	12](#_Toc234770625)

[Supplementary Figure 7: Change in neurological outcome between discharge and 12 months by diagnosis category - Unknown encephalitis	13](#_Toc234770626)

Due to the stage of their development, it was hypothesised that the full extent of neurological sequelae for young children may have been more difficult to ascertain at discharge and may manifest over time. To investigate if any age-related trends were present in the progression of neurological outcome from discharge to 12-month follow-up, the data presented in the alluvial chart shown in **Figure 1** of this report was stratified by age and causal subgroup.

The progression of outcome for each age category is shown in **Supplementary Figure 1 through** **Supplementary Figure 4**. More than half of children aged less than one with infectious encephalitis, who were categorised as having fully recovered at discharge, showed worse outcome at 12-month follow-up compared to discharge follow-up, with 1 in 4 moving from fully recovered to severe sequelae at 12 months (Supplementary Figure 1). Some children in this age group did show improvement of outcome, however no child improved by greater than one category (Supplementary Figure 1). Only one child with age greater than 10 showed worse outcome at 12 months compared to discharge (**Supplementary Figure 4**). The cause of their illness was unknown. The remaining children with age greater than 10, had similar or better outcomes at 12 months, irrespective of causal subgroup (**Supplementary Figure 4**).

Outcome progression for each causal subgroup, stratified by age category, is shown in **Supplementary Figure 5 through Supplementary Figure 7.** Infectious encephalitis (**Supplementary Figure 5)** shows greater divergence from outcome at discharge to outcome at 12 months compared to both immune mediated encephalitis (**Supplementary Figure 6**), and encephalitis of unknown aetiology (**Supplementary Figure 7**), although less so for children with age greater than 10, who only show improvement (**Supplementary Figure 5**).

Supplementary Figure 1: Change in neurological outcome between discharge and 12 months by diagnosis category - Ages 0 - 1 years

The case flows for infective, immune-mediated and unknown aetiology are shown in green, blue and red respectively. There are three nodes, the first divided into the three aetiological subgroups, and the second and third divided into the LOS severity category at discharge and at 12 months respectively. The height of the strata at the second and third node represents the number of cases in each outcome severity category, and the height of the flow at each node, represent the number of cases from each aetiology. LOS = Liverpool Outcome Score.

Supplementary Figure 2: Change in neurological outcome between discharge and 12 months by diagnosis category - Ages 1 - 5 years

The case flows for infective, immune-mediated and unknown aetiology are shown in green, blue and red respectively. There are three nodes, the first divided into the three aetiological subgroups, and the second and third divided into the LOS severity category at discharge and at 12 months respectively. The height of the strata at the second and third node represents the number of cases in each outcome severity category, and the height of the flow at each node, represent the number of cases from each aetiology. LOS = Liverpool Outcome Score.

Supplementary Figure 3: Change in neurological outcome between discharge and 12 months by diagnosis category - Ages 5 - 10 years

The case flows for infective, immune-mediated and unknown aetiology are shown in green, blue and red respectively. There are three nodes, the first divided into the three aetiological subgroups, and the second and third divided into the LOS severity category at discharge and at 12 months respectively. The height of the strata at the second and third node represents the number of cases in each outcome severity category, and the height of the flow at each node, represent the number of cases from each aetiology. LOS = Liverpool Outcome Score.

Supplementary Figure 4: Change in neurological outcome between discharge and 12 months by diagnosis category - Ages 10 to <15 years

The case flows for infective, immune-mediated and unknown aetiology are shown in green, blue and red respectively. There are three nodes, the first divided into the three aetiological subgroups, and the second and third divided into the LOS severity category at discharge and at 12 months respectively. The height of the strata at the second and third node represents the number of cases in each outcome severity category, and the height of the flow at each node, represent the number of cases from each aetiology. LOS = Liverpool Outcome Score.

Supplementary Figure 5: Change in neurological outcome between discharge and 12 months by diagnosis category - Infectious encephalitis

The case flows for each age category (>10 years, 5-10 years, 1-5 years and 0-1 years) and shown in purple, green, blue and red respectively. There are three nodes, the first divided into the age category, and the second and third divided into the LOS severity category at discharge and at 12 months respectively. The height of the strata at the second and third node represents the number of cases in each outcome severity category, and the height of the flow at each node, represent the number of cases from each age category. LOS = Liverpool Outcome Score.

Supplementary Figure 6: Change in neurological outcome between discharge and 12 months by diagnosis category - Immune-mediated encephalitis

The case flows for each age category (>10 years, 5-10 years, 1-5 years and 0-1 years) and shown in purple, green, blue and red respectively. There are three nodes, the first divided into the age category, and the second and third divided into the LOS severity category at discharge and at 12 months respectively. The height of the strata at the second and third node represents the number of cases in each outcome severity category, and the height of the flow at each node, represent the number of cases from each age category. LOS = Liverpool Outcome Score.

Supplementary Figure 7: Change in neurological outcome between discharge and 12 months by diagnosis category - Unknown encephalitis

The case flows for each age category (>10 years, 5-10 years, 1-5 years and 0-1 years) and shown in purple, green, blue and red respectively. There are three nodes, the first divided into the age category, and the second and third divided into the LOS severity category at discharge and at 12 months respectively. The height of the strata at the second and third node represents the number of cases in each outcome severity category, and the height of the flow at each node, represent the number of cases from each age category. LOS = Liverpool Outcome Score.

Appendix C: Comparative review of cases with no follow-up and those with missing data to those in the 12-month follow-up cohort.

Demographic data, clinical severity and outcome as measured by the Glasgow outcome scale (available for all patients), is shown below for the cohort with follow-up data available at 12 months (12 month follow-up cohort; N=177, Table C 1 through Table C 3), and those who were lost to follow-up (N=231, Table C 4 through Table C 6).

The proportion of cases with infectious encephalitis in the 12 month follow-up cohort was 51% compared to 63% in the cohort lost to follow-up. The distribution of other patient, clinical and outcome data was overall substantially similar between the two groups.

The most notable difference in demographic data, were the proportion of females in the immune-mediated group being under-represented in the 12 month follow-up cohort (33%, Table C 1) compared to 58% in those lost to follow-up (Table C 4). In addition, there was a higher proportion of patients in the highest socioeconomic quintile, and a lower proportion of patients whose primary language spoken at home was not English in the 12 month follow-up cohort (Table C 1).

Acute clinical severity in the follow-up cohort was similar to the full-cohort, with the most notable difference seen in ICU admission for infectious encephalitis at 57% (Table C 2) compared to 70% in the cohort lost to follow-up (Table C 4).

Patients who died or were in a persistent vegetative state at discharge did not have follow-up, hence the proportions in Table C 6 should be adjusted to those with a Glasgow outcome scale of severe disability or better, as the denominator. A lower proportion of those with severe disability participated in follow-up at 12 months, but the number of patients was low (Table C 3). The follow-up cohort had a higher proportion of cases with good recovery in the infectious (78% vs 66% [82/124,Table C 6]) and unknown (90% vs 69% [24/35, Table C 6]) causal subgroups.

Table C 1: Cohort demographics and medical history – 12 month follow-up cohort

|  | **Diagnosis Category - Encephalitis** | | |  |
| --- | --- | --- | --- | --- |
|  | **Infectious** | **Immune- mediated** | **Unknown** | **Total** |
|  | **(n=91)** | **(n=45)** | **(n=41)** | **(n=177)** |
| Female sex | 40 (44%) | 15 (33%) | 18 (44%) | 73 (41%) |
| Median age, y (IQR) | 1·6 (0·1–7·5) | 7·0 (5·3–11·2) | 6·7 (3·0–9·9) | 4·6 (0·9–9·2) |
| Age Category |  |  |  |  |
| 0–1 years | 37 (41%) | 2 (4%) | 6 (15%) | 45 (25%) |
| 1–5 years | 23 (25%) | 9 (20%) | 12 (29%) | 44 (25%) |
| 5–10 years | 19 (21%) | 18 (40%) | 13 (32%) | 50 (28%) |
| 10–15 years | 12 (13%) | 16 (36%) | 10 (24%) | 38 (21%) |
| SEIFA Quintile |  |  |  |  |
| Lowest | 19 (21%) | 8 (18%) | 8 (20%) | 35 (20%) |
| 2nd Lowest | 8 (9%) | 5 (11%) | 7 (18%) | 20 (11%) |
| Middle | 23 (26%) | 10 (22%) | 5 (12%) | 38 (22%) |
| 2nd Highest | 17 (19%) | 9 (20%) | 10 (25%) | 36 (21%) |
| Highest | 23 (26%) | 13 (29%) | 10 (25%) | 46 (26%) |
| Primary home language not English | 5/48 (10%) | 2/21 (10%) | 4/17 (24%) | 11/86 (13%) |
| Median birth weight, g (IQR) | 3230 (2900–3700) | 3244 (3000–4100) | 3500 (3100–3800) | 3300 (3000–3800) |
| Median gestational age, weeks (IQR) | 38 (38–40) | 40 (38–40) | 38 (38–40) | 39 (38–40) |
| Immunisation Status^a^ |  |  |  |  |
| Not Immunised | 6 (19%) | 0 (0%) | 1 (7%) | 7 (13%) |
| Partial | 3 (9%) | 0 (0%) | 1 (7%) | 4 (7%) |
| Up to Date | 23 (72%) | 9 (100%) | 12 (86%) | 44 (80%) |
| Not Disclosed | 0 (0%) | 0 (0%) | 0 (0%) | 0 (0%) |
| FHx Epilepsy | 16/89 (18%) | 5/43 (12%) | 7/40 (18%) | 28/172 (16%) |
| FHx MS | 7/89 (8%) | 3/43 (7%) | 2/40 (5%) | 12/172 (7%) |
| FHx Autoimmune | 19/89 (21%) | 14/43 (33%) | 8/40 (20%) | 41/172 (24%) |
| Data presented as no. (%) unless otherwise indicated | | | | |
| Abbreviations: IQR = Interquartile range; SEIFA = Socio-Economic Indexes for Areas; FHx = Family history; MS = Multiple sclerosis | | | | |
| ^a^Immunisation status as reported by the parent or guardian | | | | |

Table C 2: Overall severity of presentation - 12-month follow-up cohort

|  | **Diagnosis Category - Encephalitis** | | |  |
| --- | --- | --- | --- | --- |
|  | **Infectious** | **Immune-mediated** | **Unknown** | **Total** |
|  | **(n=91)** | **(n=45)** | **(n=41)** | **(n=177)** |
| Median hospital stay length, days (IQR) | 12 (7–17) | 13 (7–30) | 7 (4–11) | 11 (6–19) |
| ICU Admission | 52 (57%) | 13 (29%) | 19 (46%) | 84 (47%) |
| Median ICU stay length, days (IQR) | 4 (2–8) | 4 (2–8) | 1 (1–3) | 3 (1–7) |
| GCS category |  |  |  |  |
| 13–15 | 29 (53%) | 27 (73%) | 20 (57%) | 76 (60%) |
| 9–12 | 14 (25%) | 5 (14%) | 7 (20%) | 26 (20%) |
| 3–8 | 12 (22%) | 5 (14%) | 8 (23%) | 25 (20%) |
| Median number of signs and symptoms at presentation^a^ | 8 (6–11) | 10 (8–12) | 8 (7–11) | 9 (7–11) |
| Data presented as no. (%) unless otherwise indicated | | | | |
| Abbreviations: IQR = Interquartile range; ICU = Intensive Care Unit; GCS = Glasgow Coma Scale | | | | |
| ^a^Signs and symptoms list: altered level of consciousness/lethargy, decreased response to the environment, decreased eye contact, decreased arousability, seizure with loss of consciousness, personality/behaviour change, fever, focal seizure, cranial nerve abnormality, visual impairment, motor weakness, altered deep tendon reflexes, cerebellar dysfunction, abnormal movements, bladder/bowel dysfunction, irritability, headache, cough/coryza, diarrhoea, vomiting, rash | | | | |

Table C 3: Glasgow outcome scale at discharge - 12-month follow-up cohort

|  | **Diagnosis Category - Encephalitis** | | |  |
| --- | --- | --- | --- | --- |
|  | **Infectious** | **Immune mediated** | **Unknown** | **Total** |
|  | **(n=91)** | **(n=45)** | **(n=41)** | **(n=177)** |
| Glasgow Outcome Scale^a^ |  |  |  |  |
| Severe Disability | 3 (3%) | 0 (0%) | 0 (0%) | 3 (2%) |
| Moderate Disability | 17 (19%) | 15 (33%) | 4 (10%) | 36 (20%) |
| Good Recovery | 70 (78%) | 30 (67%) | 37 (90%) | 137 (78%) |
| Data presented as no. (%) | | | | |
| ^a^Assessed at discharge. One case in the infectious cohort with follow-up at 12 months, did not have GCS recorded at discharge. | | | | |

Table C 4: Cohort demographics and medical history – lost to follow-up cohort

|  | **Diagnosis Category - Encephalitis** | | |  |
| --- | --- | --- | --- | --- |
|  | **Infectious** | **Immune- mediated** | **Unknown** | **Total** |
|  | **(n=145)** | **(n=48)** | **(n=38)** | **(n=231)** |
| Female sex | 58 (40%) | 28 (58%) | 19 (50%) | 105 (45%) |
| Median age, y (IQR) | 1·3 (0·1–7·9) | 9·1 (4·5–12·5) | 6·7 (1·6–10·7) | 3·8 (0·5–9·8) |
| Age Category |  |  |  |  |
| 0–1 years | 64 (44%) | 2 (4%) | 5 (13%) | 71 (31%) |
| 1–5 years | 36 (25%) | 14 (29%) | 12 (32%) | 62 (27%) |
| 5–10 years | 20 (14%) | 13 (27%) | 10 (26%) | 43 (19%) |
| 10–15 years | 25 (17%) | 19 (40%) | 11 (29%) | 55 (24%) |
| SEIFA Quintile |  |  |  |  |
| Lowest | 43 (30%) | 9 (19%) | 10 (26%) | 62 (27%) |
| 2nd Lowest | 21 (15%) | 12 (26%) | 5 (13%) | 38 (17%) |
| Middle | 28 (20%) | 11 (23%) | 8 (21%) | 47 (21%) |
| 2nd Highest | 29 (21%) | 12 (26%) | 6 (16%) | 47 (21%) |
| Highest | 20 (14%) | 3 (6%) | 9 (24%) | 32 (14%) |
| Primary home language not English | 20 (19%) | 5 (13%) | 4 (14%) | 29 (17%) |
| Median birth weight, g (IQR) | 3000 (2700–3685) | 3500 (3100–4000) | 3525 (3000–3770) | 3210 (2860–3760) |
| Median gestational age, weeks (IQR) | 38 (37–40) | 40 (38–41) | 40 (38–40) | 39 (37–40) |
| Immunisation Status^a^ |  |  |  |  |
| Not Immunised | 12 (15%) | 0 (0%) | 0 (0%) | 12 (9%) |
| Partial | 8 (10%) | 1 (4%) | 4 (17%) | 13 (10%) |
| Up to Date | 60 (75%) | 25 (96%) | 19 (83%) | 104 (81%) |
| Not Disclosed | 0 (0%) | 0 (0%) | 0 (0%) | 0 (0%) |
| FHx Epilepsy | 15 (15%) | 4 (10%) | 4 (13%) | 23 (13%) |
| FHx MS | 1 (1%) | 1 (3%) | 2 (7%) | 4 (2%) |
| FHx Autoimmune | 7 (8%) | 9 (23%) | 7 (23%) | 23 (14%) |
| Data presented as no. (%) unless otherwise indicated | | | | |
| Abbreviations: IQR = Interquartile range; SEIFA = Socio-Economic Indexes for Areas; FHx = Family history; MS = Multiple sclerosis | | | | |
| ^a^Immunisation status as reported by the parent or guardian | | | | |

Table C 5: Overall severity of presentation – lost to follow-up cohort

|  | **Diagnosis Category - Encephalitis** | | |  |
| --- | --- | --- | --- | --- |
|  | **Infectious** | **Immune-mediated** | **Unknown** | **Total** |
|  | **(n=145)** | **(n=48)** | **(n=38)** | **(n=231)** |
| Median hospital stay length, days (IQR) | 10 (6–26) | 12 (6–33) | 10 (6–26) | 10 (6–26) |
| ICU Admission | 102 (70%) | 12 (25%) | 21 (55%) | 135 (58%) |
| Median ICU stay length, days (IQR) | 4 (2–8) | 2 (1–3) | 2 (1–4) | 3 (2–7) |
| GCS category |  |  |  |  |
| 13–15 | 35 (39%) | 30 (81%) | 14 (48%) | 79 (51%) |
| 9–12 | 26 (29%) | 5 (14%) | 8 (28%) | 39 (25%) |
| 3–8 | 29 (32%) | 2 (5%) | 7 (24%) | 38 (24%) |
| Median number of signs and symptoms at presentation^a^ | 8 (6–10) | 9 (7–11) | 9 (8–11) | 9 (7–11) |
| Data presented as no. (%) unless otherwise indicated | | | | |
| Abbreviations: IQR = Interquartile range; ICU = Intensive Care Unit; GCS = Glasgow Coma Scale | | | | |
| ^a^Signs and symptoms list: altered level of consciousness/lethargy, decreased response to the environment, decreased eye contact, decreased arousability, seizure with loss of consciousness, personality/behaviour change, fever, focal seizure, cranial nerve abnormality, visual impairment, motor weakness, altered deep tendon reflexes, cerebellar dysfunction, abnormal movements, bladder/bowel dysfunction, irritability, headache, cough/coryza, diarrhoea, vomiting, rash | | | | |

Table C 6: Glasgow outcome scale at discharge – lost to follow-up cohort

|  | **Diagnosis Category - Encephalitis** | | |  |
| --- | --- | --- | --- | --- |
|  | **Infectious** | **Immune mediated** | **Unknown** | **Total** |
|  | **(n=145)** | **(n=48)** | **(n=38)** | **(n=231)** |
| Glasgow Outcome Scale^a^ |  |  |  |  |
| Death | 18 (12%) | 0 (0%) | 3 (8%) | 21 (9%) |
| Persistent Vegetative State | 3 (2%) | 0 (0%) | 0 (0%) | 3 (1%) |
| Severe Disability | 9 (6%) | 4 (9%) | 2 (5%) | 15 (7%) |
| Moderate Disability | 32 (22%) | 13 (28%) | 9 (24%) | 54 (24%) |
| Good Recovery | 82 (57%) | 30 (64%) | 24 (63%) | 136 (59%) |
| Data presented as no. (%) | | | | |
| ^a^Assessed at discharge | | | | |

Appendix D: Univariate ordinal regression

Univariate ordinal regression was conducted on all available dependent variables to test for association with worse neurological outcomes according to the Liverpool outcome score (LOS). The results are presented in Table D 1.

Table D 1: Predictive factors for adverse neurological outcome at 12 months

| **Predictive Factor** | **n** | **Univariate OR (95% CI)** | **P Value** |
| --- | --- | --- | --- |
| Patient characteristics |  |  |  |
| Sex | 177 |  | 0·773 |
| Male |  | 1·0 (ref) |  |
| Female |  | 1·08 (0·63–1·87) |  |
| Age, years (continuous) | 177 | 0·96 (0·90–1·01) | 0·118 |
| Age Category | 177 |  | 0·245 |
| 0–1 years |  | 1·62 (0·72–3·62) |  |
| 1–5 years |  | 1·89 (0·86–4·15) |  |
| 5–10 years |  | 1·02 (0·47–2·20) |  |
| 10–15 years |  | 1·0 (ref) |  |
| SEIFA Quintile | 175 |  | 0·897 |
| Lowest |  | 1·01 (0·44–2·3) |  |
| 2nd Lowest |  | 1·25 (0·49–3·18) |  |
| Middle |  | 1·40 (0·64–3·07) |  |
| 2nd Highest |  | 1·30 (0·58–2·90) |  |
| Highest |  | 1·0 (ref) |  |
| Patient born overseas | 176 | 0·33 (0·10–1·12) | 0·061 |
| Mother born overseas | 49 | 0·45 (0·14–1·49) | 0·183 |
| Father born overseas | 49 | 0·33 (0·11–1·00) | 0·046* |
| Primary language spoken at home not English | 86 | 0·06 (0·01–0·50) | 0·001* |
| Birth Weight | 145 | NR | 0·392 |
| Gestational Age | 48 | 0·96 (0·81–1·14) | 0·646 |
| Immunisation Status | 55 |  | 0·028* |
| Not immunised |  | 6·06 (1·26–29·41) |  |
| Partially immunised |  | 4·95 (0·74–33·33) |  |
| Fully immunised |  | 1·0 (ref) |  |
| Family History |  |  |  |
| Epilepsy | 172 | 1·12 (0·54–2·34) | 0·766 |
| MS | 172 | 1·03 (0·36–2·95) | 0·960 |
| Autoimmune | 172 | 1·17 (0·62–2·19) | 0·635 |
| Clinical Severity |  |  |  |
| Hospital length of stay (days) | 177 | 1·02 (1·01–1·03) | 0·001* |
| ICU Admission | 177 | 1·53 (0·89–2·62) | 0·124 |
| ICU length of stay (days) | 84 | 1·07 (1·01–1·13) | 0·004* |
| Supplementary Oxygen | 28 | 8·26 (0·77–90·91) | 0·052 |
| CPAP used | 117 | 3·58 (0·24–55·56) | 0·217 |
| Mechanical Ventilation | 117 | 0·33 (0·04–3·03) | 0·409 |
| Inotropes administered | 177 | 2·80 (1·03–7·62) | 0.044* |
| GCS | 127 | 0·92 (0·84–1·01) | 0·083 |
| Number of signs and symptoms at   presentation^a^ | 177 | 1·09 (1–1·19) | 0·056 |
| Clinical Signs |  |  |  |
| Altered LOC/Lethargy | 175 | 0·84 (0·31–2·24) | 0·726 |
| Decreased Response to Environment | 161 | 1·51 (0·85–2·67) | 0·156 |
| Decreased Eye Contact | 150 | 1·59 (0·88–2·86) | 0·120 |
| Decreased Arousability | 170 | 1·38 (0·76–2·49) | 0·290 |
| Focal Seizure | 161 | 1·62 (0·91–2·87) | 0·101 |
| Seizure with loss of consciousness | 170 | 1·66 (0·95–2·88) | 0·073 |
| Personality/ Behaviour Change^b^ | 174 | 1·91 (1·01–3·61) | 0·045* |
| CN Abnormality | 155 | 0·84 (0·4–1·76) | 0·637 |
| Visual Impairment | 144 | 1·29 (0·62–2·70) | 0·504 |
| Motor Weakness | 163 | 2·05 (1·16–3·64) | 0·013* |
| Altered DTR | 146 | 1·36 (0·70–2·62) | 0·361 |
| Cerebellar Dysfunction | 148 | 1·29 (0·71–2·33) | 0·403 |
| Abnormal Movements | 169 | 2·09 (1·19–3·66) | 0·010 |
| Bladder/Bowel Dysfunction | 160 | 1·72 (0·94–3·16) | 0·079 |
| Fever >38C | 172 | 1·02 (0·55–1·91) | 0·942 |
| Headache | 130 | 1·07 (0·56–2·05) | 0·842 |
| Irritability | 173 | 1·04 (0·59–1·80) | 0·904 |
| Cough/Coryza | 57 | 0·61 (0·24–1·59) | 0·312 |
| Diarrhoea | 175 | 0·94 (0·48–1·86) | 0·868 |
| Vomiting | 176 | 1·12 (0·66–1·93) | 0·670 |
| Rash | 176 | 1·28 (0·68–2·40) | 0·451 |
| Investigations |  |  |  |
| CSF pleocytosis^c^ | 163 | 0·93 (0·51–1·70) | 0·812 |
| Oligoclonal bands | 71 |  | 0·356 |
| Negative |  | 1·0 (ref) |  |
| Positive |  | 0·50 (0·19–1·33) |  |
| Inconclusive |  | 0·60 (0·08–4·46) |  |
| MRI | 167 |  | 0·012* |
| Normal / Inconclusive |  | 1·0 (ref) |  |
| Consistent with encephalitis |  | 2·22 (1·19–4·13) |  |
| CT | 78 |  | 0·537 |
| Normal |  | 1·0 (ref) |  |
| Abnormal |  | 0·76 (0·31–1·88) |  |
| Inconclusive |  | 1·93 (0·40–9·43) |  |
| EEG | 98 |  | 0·489 |
| Normal / Inconclusive |  | 1·0 (ref) |  |
| Abnormal |  | 1·39 (0·55–3·50) |  |
| Aetiology |  |  |  |
| Aetiological subgroup | 177 |  | 0·638 |
| Infectious |  | 1·27 (0·65–2·48) |  |
| Autoimmune |  | 0·96 (0·44–2·10) |  |
| Unknown |  | 1·0 (ref) |  |
| Causal groups | 177 |  | 0·266 |
| Influenza |  | 1·79 (0·54–5·88) |  |
| HSV |  | 2·93 (0·88–9·80) |  |
| Parechovirus |  | 3·29 (1·04–10·42) |  |
| Enterovirus |  | 0·64 (0·22–1·83) |  |
| Mycoplasma |  | 1·23 (0·37–4·12) |  |
| Bacterial Meningoencephalitis |  | 0·74 (0·21–2·65) |  |
| Other Infectious^d^ |  | 0·85 (0·31–2·34) |  |
| ADEM |  | 0·99 (0·44–2·26) |  |
| Other Autoimmune^e^ |  | 0·84 (0·20–3·41) |  |
| Unknown |  | 1·0 (ref) |  |
| P values calculated using ordinal logistic regression; *Denotes p values < 0·05 | | | |
| Abbreviations: OR = Odds ratio; FHx = Family history; SEIFA = Socio-Economic Indexes for Areas; NR = Not reported; MS = Multiple sclerosis; ICU = Intensive care unit; CPAP = Continuous positive airway pressure; GCS = Glasgow coma scale; LOC = Level of consciousness; CN = Cranial Nerve; DTR = Deep tendon reflexes; CSF = Cerebrospinal fluid; MRI = Magnetic resonance imaging; CT = Computed tomography; EEG = Electroencephalogram; HSV = Herpes simplex virus; ADEM = Acute disseminated encephalomyelitis; NMDAR = N-methyl-D-aspartate receptor | | | |
| ^a^Signs and symptoms list: altered level of consciousness/lethargy, decreased response to the environment, decreased eye contact, decreased arousability, seizure with loss of consciousness, personality/behaviour change, fever, focal seizure, cranial nerve abnormality, visual impairment, motor weakness, altered deep tendon reflexes, cerebellar dysfunction, abnormal movements, bladder/bowel dysfunction, irritability, headache, cough/coryza, diarrhoea, vomiting, rash | | | |
| ^b^Lasting greater than 24 hours | | | |
| ^c^Defined as >15 WBC/mm³ if < 2 months old; >5 WBC/mm³ if ≥2 months | | | |
| ^d^Includes: Mixed or single causative agent including infective causes listed above and/or Adenovirus, Cryptococcus, Epstein Barr Virus, Human Herpesvirus 6, Middle Eastern Respiratory Syndrome Coronavirus, Norovirus, Parainfluenza Virus, Respiratory Syncytial Virus, Rotavirus. | | | |
| ^e^Includes: Anti-NMDAR (6), immune-mediated with unknown causative agent (2), anti-GAD (1). | | | |
